# American Society for Enhanced Recovery (ASER) and Perioperative Quality Initiative (POQI) joint consensus statement on optimal analgesia within an enhanced recovery pathway for colorectal surgery: part 1—from the preoperative period to PACU

**DOI:** 10.1186/s13741-017-0064-5

**Published:** 2017-04-13

**Authors:** Matthew D. McEvoy, Michael J. Scott, Debra B. Gordon, Stuart A. Grant, Julie K. M. Thacker, Christopher L. Wu, Tong J. Gan, Monty G. Mythen, Andrew D. Shaw, Timothy E. Miller, For the Perioperative Quality Initiative (POQI) I Workgroup, Robert H. Thiele, Karthik Raghunathan, C. S. Brudney, Dileep N. Lobo, Daniel Martin, Anthony Senagore, Stefan D. Holubar, Traci Hedrick, John Kellum, Ruchir Gupta, Mark Hamilton, S. Ramani Moonesinghe, Mike P. W. Grocott, Elliott Bennett-Guerrero, Thomas J. Hopkins, Roberto Bergamaschi, Stuart McCluskey, Vijaya Gottumukkala

**Affiliations:** 1 Department of Anesthesiology, CIPHER (Center for Innovation in Perioperative Health, Education, and Research) Vanderbilt University Medical Center, 2301VUH, Nashville, TN 37232 USA; 2 Anaesthesia & Intensive Care Medicine, Royal Surrey County NHS Foundation Hospital, Surrey, UK; 3 Department of Anaesthesia, University of Surrey, Surrey, UK; 4 University College London, London, UK; 5 Harborview Integrated Pain Care Program, Department of Anesthesiology & Pain Medicine, University of Washington, Seattle, USA; 6 Division of Regional Division, Department of Anesthesiology, Duke University Medical Center, Durham, USA; 7 Division of Advanced Oncologic and GI Surgery, Department of Surgery, Duke University Medical Center, Durham, USA; 8 Department of Anesthesiology/Critical Care Medicine, The Johns Hopkins University School of Medicine, Baltimore, USA; 9 Department of Anesthesiology, Stony Brook University School of Medicine, Suffolk, USA; 10 UCL/UCLH National Institute of Health Research Biomedical Research Centre, London, UK; 11 Department of Anesthesiology, Vanderbilt University, Nashville, USA; 12 Division of General, Vascular and Transplant Anesthesia, Duke University Medical Center, Durham, USA

**Keywords:** Enhanced recovery pathway, Colorectal, Optimal analgesia, Pain management, Multimodal, Non-opioid adjuncts, Perioperative, Outcomes, Quality

## Abstract

**Background:**

Within an enhanced recovery pathway (ERP), the approach to treating pain should be multifaceted and the goal should be to deliver “optimal analgesia,” which we define in this paper as a technique that optimizes patient comfort and facilitates functional recovery with the fewest medication side effects.

**Methods:**

With input from a multi-disciplinary, international group of clinicians, and through a structured review of the literature and use of a modified Delphi method, we achieved consensus surrounding the topic of optimal analgesia in the perioperative period for colorectal surgery patients.

**Discussion:**

As a part of the first Perioperative Quality Improvement (POQI) workgroup meeting, we sought to develop a consensus document describing a comprehensive, yet rational and practical, approach for developing an evidence-based plan for achieving optimal analgesia, specifically for a colorectal surgery ERP. The goal was two-fold: (a) that application of this process would lead to improved patient outcomes and (b) that investigation of the questions raised would identify knowledge gaps to aid the direction for research into analgesia within ERPs in the years to come. This document details the evidence for a wide range of analgesic components, with particular focus from the preoperative period to the post-anesthesia care unit. The overall conclusion is that the combination of analgesic techniques employed in the perioperative period is not important as long as it is effective in delivering the goal of optimal analgesia as set forth in this document.

## Introduction

Pain after major abdominal surgery is severe and is a major component of the stress response if not adequately treated (Schricker and Lattermann 2015). Pain is triggered as a combination of neural and inflammatory pathways with injury to the viscera, muscle, and skin. The intensity and duration of each of these triggers varies according to the type of surgical procedure performed and which surgical approach is used (laparoscopic, robotic assisted, or open) (Reza et al. 2006). Additionally, patients respond differently to pain in the perioperative period and patients with chronic pain conditions often experience a greater amount of suffering in the immediate perioperative period. Thus, it is not only important to treat pain effectively from a humane point of view but also because this is a major factor in reducing the stress response to surgery and restoring function thereafter.

**Fig. 1:**
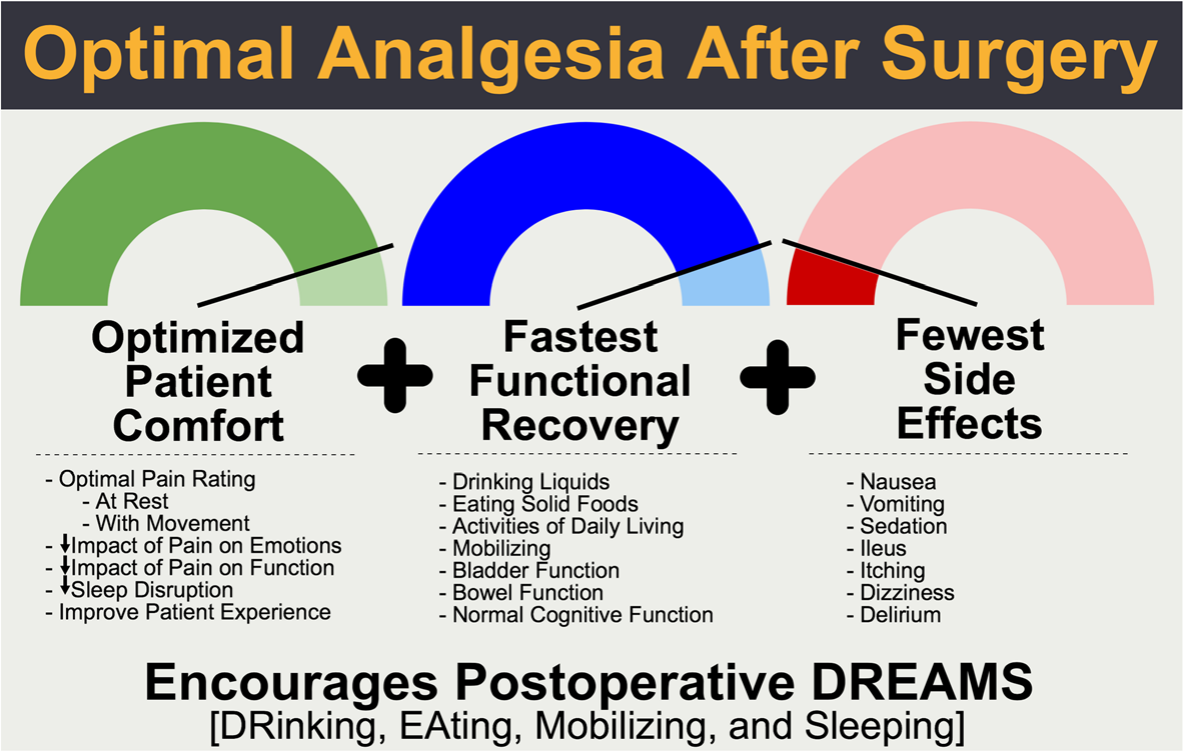
The core components of providing optimal analgesia. Pain after surgery can have profound effects on patient recovery. However, the complete elimination of pain may also have untoward effects, as listed in the figure. Optimal analgesia after surgery is an approach to pain control that facilitates a positive patient experience through optimized patient comfort that facilitates functional recovery while minimizing adverse drug events

The concept of an enhanced recovery pathway (ERP) is a multi-component approach aimed at reducing the stress of surgery experienced by the patient, improving the metabolic response, and thereby speeding the return of functional recovery (Kehlet and Wilmore 2008). Within an ERP, the approach to treating pain should be multifaceted, including a combination of techniques such as neural blockade, intravenous, and multimodal oral analgesia. The goal should be to deliver “optimal analgesia,” which we define in this paper as a technique that optimizes patient comfort and facilitates functional recovery with the fewest medication side effects (see Fig. 1). Of note, this may not correspond with the lowest pain perception possible. Overall, the combination of analgesic techniques employed is not important as long as it is effective in delivering this goal of optimal analgesia.

As a part of the first Perioperative Quality Improvement (POQI) workgroup meeting, we sought to develop a consensus document addressing these questions. Our intent was to develop a comprehensive, yet rational and practical, approach for developing an evidence-based plan for achieving optimal analgesia specifically for a colorectal surgery (CRS) ERP. The goal would be two-fold: (a) that application of this process would lead to improved patient outcomes and (b) that investigation of the questions raised would identify knowledge gaps to aid the direction for research into analgesia within ERPs in the years to come. The overall vision for our working group was to encourage rigorous development and application of evidence-based perioperative medicine related to achieving optimal analgesia for patients undergoing CRS.

## Methods

We used the Delphi method to achieve consensus surrounding the topic of optimal analgesia in the perioperative period for colorectal surgery patients. The Delphi method has been used in various formats to obtain the perspectives and opinions of diverse groups. The participants in the POQI consensus meeting included anesthesiologists, surgeons, and nurses who were recruited based on their expertise in the principles of enhanced recovery after surgery and perioperative medicine. For our use, the process included several iterative steps, including building consensus around the important questions related to the topic, a literature review of the topics, and sequential steps of content building and refinement until agreement is achieved and a consensus document is produced.

### Expert group and process

A group of international experts was established, including viewpoints representing anesthesiology, surgery, and nursing. In this POQI I subgroup, each expert was required to submit questions related to optimal perioperative analgesia within ERPs. This first project specifically focused on colorectal surgery patients. Questions were then shared among the group for commentary and elaboration. A final list of questions was agreed upon by the end of the 2-day conference after undergoing a four-step modified Delphi process (Miller et al. 2016).

For content to be included in the paper, we searched PubMed from 1966 to April 2016. All co-authors were familiar with proper literature search protocols, and each conducted a search for at least one portion of the consensus document and shared those references with the other experts. The search was limited to human trials but not limited by language. Duplicate records were deleted. The authors screened the search results in a stepwise manner to identify the eligible studies. In the first step, we screened the titles and abstracts, and irrelevant papers were excluded. During the POQI I conference and thereafter as a writing group, reference applicability to the topic was discussed in any area where there was disagreement.

## Results

Our group arrived at the following list of questions as being those most pertinent to and all-encompassing of the topic of optimal analgesia as a component of an ERP for CRS:

1. What is the definition of optimal analgesia for colorectal surgery?
2. Why should opioid use be minimized for colorectal surgery patients?
3. How can optimal analgesia be achieved whilst minimizing opioid use in the preoperative and intraoperative period for colorectal surgery?
4. How does pain vary based upon the *surgical approach in colorectal surgery*?
5. What strategies lead to successful implementation of optimal analgesia for colorectal surgery?

### Q1: What is the definition of optimal analgesia for colorectal surgery?

Statement: Optimal analgesia can be defined as a technique that optimizes patient comfort and facilitates recovery of physical function including the bowel, mobilization, cough and normal sleep, while minimizing adverse effects of analgesics (see Fig. 1).

Optimal analgesia cannot be defined by simple pain intensity ratings. Although pain intensity ratings have been associated with impairments in function, there is a nonlinear relationship between types of treatment, analgesic doses, and changes in self-reported numeric pain ratings (de C Williams et al. 2000). Pain after surgery is rarely completely avoided, so “pain free” is not the primary goal of optimal analgesia. Additionally, pain over hours to days after surgery is a dynamic state that is influenced by activities such as coughing and ambulation, and thus a pain-free state is difficult to attain and sustain without medication side effects. Accordingly, the goal of pain prevention and treatment is to reduce pain interference on surgical recovery to the greatest degree possible and avoid secondary adverse outcomes evoked by inflammation, the stress response, and immobilization; hence, the emphasis of optimal analgesia on patient comfort combined with considerations of physical function, sleep, side effects, and safety.

### Q2: Why should opioid use be minimized for colorectal surgery patients?

Statement: Minimizing opioid analgesia for CRS patients reduces the adverse effects of opioid use.

There are some clear governing principles that can guide pain management planning to minimize opioid use, and, as discussed above, numerous interventions exist to aid in this approach while optimizing perioperative analgesia. However, opioids have been the backbone for treating perioperative pain and are still used extensively, if not exclusively, in most surgical specialties. As such, it should be noted that the short-term side effects of opioids including nausea, vomiting, ileus, urinary retention, and somnolence can delay enteral intake and mobilization, cause patient distress, and delay hospital discharge in the colorectal surgical patient. Additionally, postoperative delirium in the elderly is a frequent complication that delays discharge and can be caused both by uncontrolled pain and its treatment with opioids (Oresanya et al. 2014; Bilotta et al. 2013; Leung et al. 2013). Finally, traditional use of an opioid-based pain management regimen is likely to be associated with developing hyperalgesia, whereas the use of non-opioid approaches may result in reduced chronic postsurgical pain, cancer recurrence, and long-term survival (Hayhurst and Durieux 2016; Maher and White 2016). This has led to the adoption of a multifaceted approach using various analgesic components to greatly reduce the need to give any opioids during the perioperative period. For all the reasons detailed above, opioid-sparing pathways should be considered a best practice.

### Q3: How can optimal analgesia be achieved while minimizing opioid use in the preoperative and intraoperative period for colorectal surgery?

Statement: Optimal analgesia after CRS is achieved through a planned multimodal analgesia approach minimizing opioid use during all phases of perioperative care.

**Fig. 2:**
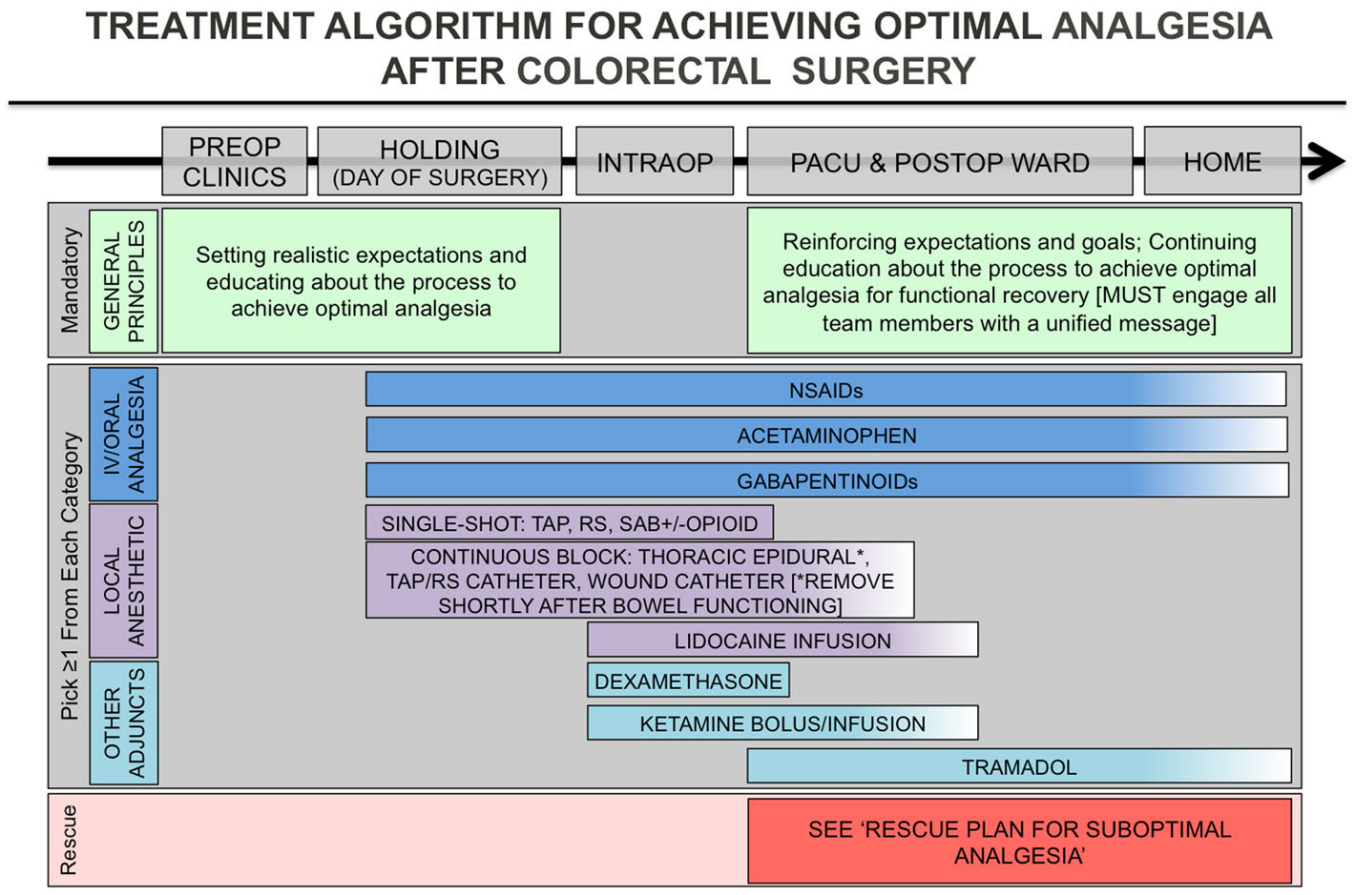
Suggested components of a multimodal approach to pain management in an ERP for colorectal surgery. Of note, the plan should be comprehensive, encompassing all phases of perioperative care from preoperative to post-discharge. However, current evidence is insufficient to determine how many components should be selected in order to maximize pain control, reduce opioid burden, and avoid the side effects of all analgesics used. (*ERP* enhanced recovery pathway)

In order to deliver optimal analgesia, a well-structured and planned multimodal approach should be constructed that spans from the preoperative period into the post-discharge recovery phase (see Fig. 2). The components of such a plan will be discussed in detail in this manuscript. It should be noted that there are numerous successful ERPs for CRS with similar but varying analgesic components represented in Fig. 2 (Thiele et al. 2015; Miller et al. 2014; Larson et al. 2014; McEvoy et al. 2016). What is known is that reducing opioid use is of benefit, and high compliance with a variety of standardized non-opioid ERP bundles is strongly associated with reduced opioid use and improved outcomes. In this document, we will restrict the discussion to preoperative and intraoperative components. Part 2 will discuss the analgesic approach throughout all phases of postoperative care (Scott et al. 2016).

#### Preoperative interventions

##### Neural blockade

Use of a single-shot spinal opioid (i.e., morphine or hydromorphone) is associated with significantly lower pain at rest and on movement, and reduced opioid requirements (Meylan et al. 2009). These benefits are more prominent in patients undergoing abdominal versus other types of surgery (e.g., cardiac). Although the dose range for this meta-analysis varies considerably (dose range, 100–4000 mcg), current practice tends toward using lower doses of intrathecal morphine (<0.3 mg) as higher dose of intrathecal morphine are associated with more episodes of respiratory depression (Gehling and Tryba 2009). Some centers use a spinal dose of bupivacaine as a carrier for the opioid to cover the incision although there is often a resulting sympathetic block.

Use of thoracic epidural anesthesia (TEA) for open CRS is associated with superior postoperative analgesia (Werawatganon and Charuluxanun 2005; Block et al. 2003), decreased pulmonary/cardiac morbidity (Popping et al. 2014), and earlier return of gastrointestinal function as compared to parenteral analgesia (Marret et al. 2007; Hughes et al. 2014). However, the overall benefits of TEA in improving recovery or decreasing length of stay in patients undergoing laparoscopic colorectal procedures are uncertain (Liu et al. 2014; Khan et al. 2013). Concern that prolonged sympathetic blockade in TEA requires patients to have further intravenous fluids to maintain intravascular volume in face of arterial hypotension were not observed in one meta-analysis of TEA versus patient-controlled analgesia (PCA) in laparoscopic colectomy (Liu et al. 2014).

##### Peripheral blocks: truncal, paravertebral, and surgical site

Peripheral regional analgesia options for CRS patients include transversus abdominis plane (TAP), paravertebral, or wound and peritoneal infiltration blocks/catheters. All have been shown to some extent to improve perioperative analgesia while decreasing opioid use. Paravertebral blocks and catheters for surgical anesthesia at the level of the thoracic and lumbar vertebrae are associated with less pain during the immediate postoperative period (Thavaneswaran et al. 2010).

Multiple meta-analyses indicate that TAP blocks/catheters for abdominal surgical procedures are associated with superior analgesia and decreased postoperative opioid consumption compared to opioid analgesia alone (Baeriswyl et al. 2015; Zhao et al. 2014; Johns et al. 2012; Siddiqui et al. 2011; Charlton et al. 2010). Preoperative (vs. postoperative) TAP block administration appears to have greater effects on early pain and opioid consumption compared with postoperative administration although the effect of preoperative (vs. postoperative) TAP blocks on longer-term outcomes is unknown (De Oliveira et al. 2014). TAP blocks provide comparable short-term analgesia to wound infiltration but provide superior analgesia in longer term and in the setting of a multimodal analgesic regimen (Yu et al. 2014; Guo et al. 2015a). Concerning the choice of local anesthetic, ropivacaine, bupivacaine, and liposomal bupivacaine have all been used in ERPs with good results (Hamada et al. 2016; Cohen 2012). Liposomal bupivacaine shows some promise for longer-term postoperative analgesia either as infiltration or for TAP blocks (Hutchins et al. 2015); however, there are no large-scale randomized controlled trials available in intra-abdominal surgery to guide practice or to definitively demonstrate the analgesic efficacy of this intervention (Cohen 2012; Candiotti et al. 2014). In short, while there is insufficient evidence to recommend one medication over another at this point in time, it should be noted that additives, such as dexamethasone, are needed to prolong the duration of non-liposomal mixtures (Akkaya et al. 2014).

Intraperitoneal instillation of local anesthetics during major abdominal surgery, including open and laparoscopic colectomy, is associated with significantly lower pain scores postoperatively; although one study did continue TEA in addition to intraperitoneal instillation for 2 days after surgery (Marks et al. 2012; Kahokehr et al. 2011; Park et al. 2011). Additionally, wound infiltration has been shown to be associated with a decrease in morphine consumption and significantly lower pain scores in the early postoperative period in abdominal surgery patients, but none specifically in colorectal surgery (Bamigboye and Hofmeyr 2009). A meta-analysis suggested that the use of local anesthetic wound infiltration was associated with pain scores comparable to those obtained with epidural analgesia and a slight decrease in opioid use, although the data was noted to be quite heterogeneous and significance might exist in the patients receiving each treatment (Ventham et al. 2013). The analgesic efficacy of local anesthetic infusion through wound catheters is uncertain, and meta-analyses have been conflicting in CRS (Liu et al. 2006; Gupta et al. 2011).

##### Oral analgesia

Major non-opioid oral analgesic agents include non-steroidal anti-inflammatory agents (NSAIDs), acetaminophen (paracetamol), gabapentinoids (gabapentin and pregabalin), and tramadol. All except oral tramadol have been examined within the setting of intra-abdominal surgery and have been found to have significant effect on reducing the opioid burden postoperatively. As such, routine, scheduled use of these agents should be considered as part of a plan to achieve optimal analgesia after colorectal surgery.

**Acetaminophen (paracetamol)**

Acetaminophen (paracetamol) when administered as part of a multimodal regimen is associated with a decrease in pain and decrease in opioid usage, which may result in a decrease in some opioid-related side effects (Doleman et al. 2015; De Oliveira et al. 2015; Wong et al. 2013; Apfel et al. 2013; McNicol et al. 2011; Toms et al. 2008; Remy et al. 2005). A single dose of IV acetaminophen (paracetamol), typically in a dose of 1 g, given prior to surgery (meta-analysis of 11 RCTs of 740 patients) was associated with significantly lower early pain at rest, early pain with movement, postoperative opioid consumption, and postoperative nausea and vomiting (De Oliveira et al. 2015). In a meta-analysis of 7 RCTs (*n* = 544 participants), 1 g or 15 mg/kg of IV acetaminophen (paracetamol) given 10–30 min before induction/incision (vs. the same dose given 10–30 min at the end of surgery/before skin closure) was associated with a reduction in 24-h opioid consumption and a lower incidence of postoperative vomiting in the preventive acetaminophen (paracetamol) group (Doleman et al. 2015). Most studies including pharmacokinetic outcomes reported higher postoperative plasma concentrations and larger proportions of patients achieving target plasma concentrations after IV dosing compared with oral dosing (Jibril et al. 2015). However, for patients who can take oral medications preoperatively, there does not appear to be evidence of a clear benefit of the intravenous formulation. Decision making should take into account of convenience and cost (Jibril et al. 2015).

**Non-steroidal anti-inflammatory drugs**

NSAIDs, whether non-selective or cyclooxygenase-2 inhibitors (COX-2), when administered as part of a multimodal regimen, are associated with a decrease in pain and decrease in opioid usage which may result in a decrease in some opioid-related side effects (De Oliveira et al. 2012; Marret et al. 2005; Straube et al. 2005; Maund et al. 2011; Michelet et al. 2012; Elia et al. 2005). Use of COX-2 inhibitors has minimal effect on coagulation even at supra-therapeutic doses (Leese et al. 2000). A systematic review noted that preoperative COX-2 inhibitors significantly reduced postoperative pain, analgesic consumption, and antiemetic use, and improved patients satisfaction compared with preoperative placebo (Straube et al. 2005). In the studies examining celecoxib, the doses used were 200 or 400 mg PO, and for parecoxib, they were 40 mg PO [Pandazi, 2010].

It is uncertain whether the perioperative use of NSAIDs carries a risk of harm. While it is unlikely that NSAIDs increase the risk of renal injury in euvolemic patients who do not have contraindications to receiving these medications (Myles and Power 1998), caution should be undertaken in patients who are hypotensive or thought to be hypovolemic. Additionally, there is a concern for the potential for an association with increased anastomotic leak, but the literature surrounding this question is not conclusive. As such, insufficient evidence is available to recommend against routine use of NSAIDs, especially COX-2 inhibitors, as these medications are effective in treating pain and reducing opioid use in the perioperative period (Chou et al. 2016a; Bhangu et al. 2014).

**Gabapentinoids**

Several meta-analyses including studies concerning intra-abdominal surgery suggest that gabapentinoids (gabapentin, pregabalin) when given as a single dose preoperatively are associated with a decrease in postoperative pain and opioid consumption at 24 h (Engelman and Cateloy 2011; Eipe et al. 2015; Hurley et al. 2006; Mishriky et al. 2015; Peng et al. 2007; Seib and Paul 2006; Zhang et al. 2011). For gabapentin, a preoperative dose of 300–1200 mg is associated with lower pain scores (both at rest and with movement) and reduced opioid consumption (Hurley et al. 2006; Peng et al. 2007; Seib and Paul 2006). It should be noted that one small RCT found that a single preoperative dose of gabapentin 600 mg PO did not significantly reduce opioid consumption or pain scores on POD 1 or 2 for patients presenting for colectomy (Siddiqui et al. 2014). However, opioid consumption and pain scores were lower at all time points in the gabapentin group compared to placebo, but there were only 36 patients per group and it was underpowered to detect any difference. As noted by the authors, continuing doses in the postoperative period may confer added benefit given the pharmacokinetics of gabapentin. This corresponds with the dosing reported in successful ERPs for CRS where gabapentin is used as one component to reduce opioid consumption in the perioperative period (Larson et al. 2014; McEvoy et al. 2016). However, the exact contribution of gabapentin to these positive outcomes is unknown. For pregabalin, a recent meta-analysis indicated that pain scores at rest were reduced with all doses of pregabalin (mostly 75–300 mg) but pain scores with movement were only reduced with the 300 mg dose and there were no significant differences in side effects between the three dose levels of pregabalin. The opioid-sparing effect of pregabalin appeared to be limited to doses 100–150 and 300 mg but not ≤75 mg at 2 h after surgery (Mishriky et al. 2015). While most of the studies in the meta-analysis involve abdominal hysterectomy and cholecystectomy, none were in CRS patients. Of note, there is a substantial cost difference at present between gabapentin and pregabalin. As such, if a gabapentinioid is to be considered as one component in an ERP, we recommend use of gabapentin as a first line agent unless the patient was prescribed pregabalin for a chronic pain condition prior to surgery or if gabapentin use resulted in significant sedation.

#### Intraoperative

##### Intravenous medications

**Lidocaine**

Intravenous (IV) lidocaine infusion is indicated as part of a multimodal analgesic approach for visceral surgery when other local anesthetic approaches such as regional analgesia are not possible. In open and laparoscopic abdominal surgery, IV lidocaine infusions have been shown to result in significant reduction in postoperative pain intensity at rest and with cough and movement and opioid consumption for up to 48 h postoperatively, as well as being associated with earlier return of bowel function allowing for earlier recovery and shorter length of stay (Marret et al. 2008; Vigneault et al. 2011). Lidocaine infusions are contraindicated in patients with cardiovascular instability and concomitant use of alpha agonists or beta-blockers and in patients with allergies to other amide local anesthetics (bupivacaine). Side effects are more pronounced in patients with liver dysfunction, pulmonary diseases when the predominant problem is carbon dioxide retention, and congestive heart failure. Lidocaine is typically administered as a bolus (100–150 mg or 1.5–2.0 mg/kg) followed by an infusion of 1 to 3 mg/kg/h through the end of surgery. Several meta-analyses suggest that perioperative administration of IV lidocaine is associated with a decrease in postoperative pain and opioid consumption and possibly faster return of bowel function and decreased length of hospital stay (Marret et al. 2008; Vigneault et al. 2011; Khan et al. 2016; McCarthy et al. 2010).

***N*** **-methyl-** **d** **-aspartate antagonists**

***Ketamine***. Perioperative inhibition of *N*-methyl-d-aspartate (NMDA) receptors with clinically available NMDA antagonists such as ketamine may be associated with improved perioperative pain and decreased opioid use (Wang et al. 2016; Ding et al. 2014; Dahmani et al. 2011; Bell et al. 2006). Perioperative ketamine, including boluses as well as intraoperative and postoperative low-dose infusions for up to 48 h, has been shown to result in significant reductions in pain, opioid consumption, and PONV with no significant side effect profile (Zakine et al. 2008; Laskowski et al. 2011; Sami Mebazaa et al. 2008). The intraoperative boluses ranged from 0.15 to 1 mg/kg, and perioperative infusions ranged from 1 to 5 mcg/kg/min, with a postoperative infusion rate of 2 mcg/kg/min. Ketamine has also been shown to be of particular benefit in patients on chronic opioid, but this has not been specifically tested in chronic pain patients undergoing CRS (Loftus et al. 2010).

***Magnesium***. Systemic infusions of perioperative magnesium may reduce postoperative pain and opioid consumption (De Oliveira et al. 2013; Guo et al. 2015b; Murphy et al. 2013). The optimal dosing is uncertain as some studies include both a bolus followed by an infusion whereas others only utilize an infusion without a loading bolus. Typical boluses are 30–50 mg/kg, and the infusion rates range from 4 to 15 mg/kg/h. None of the studies in a systematic review reported clinical toxicity related to toxic serum levels of magnesium (De Oliveira et al. 2013).

**Glucocorticoids**

Glucocorticoid steroids may have analgesic properties possibly related to anti-inflammatory properties and should be considered as part of a multimodal perioperative pain regimen. Several meta-analyses examined perioperative dexamethasone and indicated that patients who received dexamethasone (4–10 mg or >0.1 mg/kg) had lower pain scores, used less opioids, and required less rescue analgesia (Waldron et al. 2013; Allen et al. 2012; De Oliveira et al. 2011). The concern for significant hyperglycemia (>180 mg/dL) has not been confirmed, even in bariatric patients receiving these doses of dexamethasone (Hans et al. 2006).

**Alpha**-**2 agonists**

A Cochrane review of dexmedetomidine infusions for pain found reduced opioid consumption but no significant difference in pain scores compared to placebo, and there was more hypotension in the dexmedetomidine group (Jessen Lundorf et al. 2016). Dexmedetomidine has been added both perineurally to nerve blocks and intravenously to prolong nerve block (Abdallah et al. 2016; Das et al. 2016). There is limited data, but dexmedetomidine does appear to prolong nerve blocks. However, the extended duration is not as long as that provided by perineural dexamethasone provides. In a recent extensive review of perioperative alpha agonists, dexmedetomidine and clonidine were compared (Blaudszun et al. 2012). Similar to dexmedetomidine, clonidine can also reduce opioid consumption. In addition, both alpha agonists appear to have a weak antiemetic effect, but as expected, both drugs had adverse effects on hemodynamics. In summary, both dexmedetomidine and clonidine when administered perioperatively can reduce morphine consumption up to 24 h and to a similar extent as acetaminophen (paracetamol), but not as much as other NSAIDs. Both clonidine and dexmedetomidine have other side effects such as sedation and hypotension that have to be considered (Garg et al. 2014). Typical doses of clonidine range from 1 to 5 mcg/kg PO, IV, or perineurally and for dexmedetomidine from 0.5 mcg/kg IV bolus followed by an infusion of 0.2–0.7 mcg/kg/h or 0.5 mcg/kg perineurally.

**Acetaminophen (paracetamol)**

If oral acetaminophen (paracetamol) is administered preoperatively as part of a multimodal analgesia pathway, there is typically no need to administer it again until the next scheduled dose. If the next scheduled dose is possible orally, then administration should be oral. However, if the preoperative dose has been missed or oral administration is not possible when the next dose is due, then intravenous acetaminophen (paracetamol) can be administered. Currently, there is no evidence that intravenous acetaminophen (paracetamol) is superior to oral formulations as an analgesic (Jibril et al. 2015; Fenlon et al. 2013). If a patient is not able to take oral medications, then intravenous acetaminophen (paracetamol) has been shown to be an effective opioid-sparing analgesic compared to placebo (O’Neal 2013; Smith 2011).

**Non**-**steroidal anti**-**inflammatory drugs**

As noted above, NSAIDs should be prescribed orally as part of routine preoperative medications for colorectal surgery. However, if a preoperative dose is not given for concerns of bleeding, parenteral options are available. Ketorolac has been used extensively, and newer preparations including intravenous ibuprofen and diclofenac are now available (De Oliveira et al. 2012; Kroll 2012). These medications can improve pain scores, reduce opioid requirements, and reduce opioid-related side effects as discussed in the section above. The optimal dose and timing of each of these medications is not yet known. There is currently no evidence of a better risk benefit profile for one intravenous NSAID over another.

**Tramadol**

Overall, there is limited evidence for the use of tramadol in colorectal surgery. However, one study in particular compared postoperative pain control with an opioid IV-PCA to IV tramadol and found that the patients in the non-PCA (tramadol) group needed less rescue analgesia and none of them needed to have an IV-PCA started (Choi et al. 2015). Additionally, another study included scheduled tramadol as part of an ERP (Lloyd et al. 2010). While this was only one component of the ERP, overall pain scores and other outcomes were improved. If tramadol is to be used, one study would suggest caution with its use in patients undergoing major abdominal surgery who are over 75 years, ASA 3 or 4, and have impaired mobility or frailty, as use in this setting was associated with delirium (Brouquet et al. 2010). Based on the evidence that exists in colorectal surgery, we recommend considering tramadol as an analgesic adjunct as one part of an ERP.

**Opioids**

The primary objective of the practitioner should be to minimize the use of opioids wherever possible. Under general anesthesia, patients are unconscious and do not perceive pain. Although there is no pain perception, surgery-induced activation of nociceptive reflex arcs has hemodynamic consequences. Using opioids to reduce the hemodynamic changes should be resisted as much as possible, as hyperalgesia can develop acutely and opioid-related side effects can occur at very low doses (Hayhurst and Durieux 2016; Rathmell et al. 2006). Control of blood pressure and heart rate at intubation and during surgery by beta-blockers, calcium channel blockers, or lidocaine should be encouraged rather than using opioids without a specific indication other than intraoperative tachycardia or hypertension. As noted above, replacing opioids with any non-opioid analgesic or esmolol intraoperatively leads to *less* opioid being required in the PACU and often out to 24 h postoperatively in some laparoscopic surgery (Lee and Lee 2010; Collard et al. 2007; Pranevicius and Pranevicius 2009). If an opioid must be used, we recommend using small doses of short-acting opioids such as alfentanil or fentanyl.

### Q4: How does pain vary based upon the *surgical approach in colorectal surgery*?

Statement: The degree of pain after CRS will vary based upon the surgical approach and planned analgesic solutions will take this into account.

As the pain experience is different based on the procedure, so too should the analgesia be procedure and patient-specific. While not considered an analgesic strategy, a number of studies have reported reduction in short-term pain, analgesic use, and more rapid recovery of bowel function with laparoscopic surgery versus open (Reza et al. 2006). The difference in the pain experience between laparoscopic and open approaches for operations may be limited to the short-term management, as pain reported at 1 to 3 months did not differ between approaches (Reza et al. 2006; Lourenco et al. 2008; Murray et al. 2006). One important difference in laparoscopic CRS compared to other laparoscopic procedures is the placement of ports and size and placement of the largest incision. Different from a gallbladder or a morselized uterus, the colorectal specimens have to be removed intact. Often, the bowel anastomosis is most easily organized extra-corporeally as well. This longer incision, for specimen extraction and anastomosis, may be umbilical, transverse, upper or lower midline, or Pfannenstiel. Overall, the chosen approach/incision and its length certainly impact the best choice of analgesia plan. Additionally, as the surgeon can occasionally choose to vary approach intraoperatively as needed, a secondary plan should be in place for these circumstances.

### Q5: What is the role of education in achieving optimal analgesia after CRS?

Statement: Patient and family education throughout the entire perioperative period is essential for achieving optimal analgesia after CRS.

Optimal analgesia cannot be achieved without involving the most important factor, the patient, starting with partnership, education, shared decision making, and well-coordinated transitions of care (Manary et al. 2013). Because pain is a complex and subjective biopsychosocial experience, management of patient expectations and education regarding realistic goals of pain treatment is crucial to an effective pharmacological approach. Thus, we recommend this includes analgesic treatment plans and goals for postoperative pain management (Wood 2010; Oshodi 2007a; Oshodi 2007b). While the exact timing, methods, and content of preoperative education will be locally determined, we suggest that patient education and expectation management occur through all phases of care and include information about choice and risks of analgesic technique, goals of analgesia, anticipated patient participation in recovery activities, and non-pharmacological methods that can be employed to reduce reliance on rescue analgesics (see Fig. 2) (Chou et al. 2016b; O’Connor et al. 2014). Important considerations include information about analgesia methods and goals in patient-friendly print materials with appropriate literacy levels (Ihedioha et al. 2013).

Additionally, as some patients will have had surgery prior to the institution of an ERP, and as ERPs for CRS typically utilize a multimodal analgesic approach to minimize perioperative opioid use in an attempt to decrease opioid-related side effects, it is important to discuss surgical history and pain management expectations based on prior interventions and experiences. This is especially true as some ERPs may attempt to avoid IV-PCA opioids and thus use opioids only as PRN rescue, as recommended in Part 2 (McEvoy et al. 2016; Wu et al. 2015). It should be explained to patients that opioids still remain an important option for postoperative pain management; however, the precise role of opioids in ERPs is currently not clearly defined, and expectations surrounding their use in the perioperative, along with the goals of delivering optimal analgesia, should be clarified preoperatively.

## Summary and future directions

Delivering optimal analgesia is a key component of enhanced recovery pathways for colorectal surgery. Following our literature search and modified Delphi process, we conclude that the number of published studies specifically examining analgesia for CRS within an ERP with good documented compliance is limited. We have used data from these studies and extrapolated data from other published studies on analgesia for CRS to recommend a best-practice approach for creating a perioperative pain management plan that includes a number of key components (see Table 1).

**Table 1:** Key points for analgesia within an ERP for colorectal surgery

• Analgesia is a key component in enhanced recovery pathways.	
• Optimal analgesia addresses patient pain while restoring function and minimizing side effects.	
• Minimizing opioid use and its side effects is a cornerstone of analgesia practice within ERPs.	
• Intraoperative opioid-sparing techniques and postoperative early oral multimodal analgesia are the backbone for providing analgesia within ERPs.	
• Open, laparoscopic, and robotic surgical approaches need different analgesic strategies.	
• There are many different analgesic combinations that are efficacious.	
• Hospitals should adopt at least two or three analgesic strategies for colorectal surgery to allow for individual patient variation or failure of the primary choice of analgesia.	
• Hospitals should have a troubleshooting pathway in place for breakthrough pain to minimize the negative impact of intravenous opioid use.	
• Audit of compliance of analgesia and restoration of function can lead to improvement of patient experience.	

There are many ways to achieve analgesia for patients undergoing CRS. The technique of choice will depend on which surgical procedure is performed and by which approach (laparoscopic, robotic assisted, or open). Additionally, patient factors and availability of experienced providers, training, and equipment within a hospital will also determine which technique can be utilized. A multifaceted approach to pain management is necessary to restore postoperative function as rapidly as possible to baseline. Importantly, this must facilitate mobility and respiratory function, enable early oral intake of diet and hydration, avoid ileus, promote normal sleep patterns, and minimize the common side effects of opiate-based analgesia. We have produced a consensus outlining a rationale and key components for approaching pain management in an ERP for CRS. Part 2 of this series will discuss the postoperative components of an optimal analgesia care plan for CRS.

## Abbreviations

COX-2: Cyclooxygenase-2
CRS: Colorectal surgery
ERP: Enhanced recovery pathway
IV: Intravenous
NMDA: *N*-methyl-d-aspartate
NSAIDs: Non-steroidal anti-inflammatory agents
PACU: Post-anesthesia care unit
PCA: Patient-controlled analgesia
PONV: Postoperative nausea and vomiting
POQI: Perioperative Quality Improvement
TAP: Transversus abdominis plane
TEA: Thoracic epidural analgesia
